# Characterization of the novel tumor-suppressor gene *CCDC67* in papillary thyroid carcinoma

**DOI:** 10.18632/oncotarget.6709

**Published:** 2015-12-22

**Authors:** De-Tao Yin, Jianhui Xu, Mengyuan Lei, Hongqiang Li, Yongfei Wang, Zhen Liu, Yubing Zhou, Mingzhao Xing

**Affiliations:** ^1^ Department of Thyroid Surgery, The First Affiliated Hospital of Zhengzhou University, Zhengzhou, 450052, P. R. China; ^2^ Key Discipline Laboratory of Clinical Medicine Henan, Zhengzhou 450052, P. R. China; ^3^ Department of Pharmacy, The First Affiliated Hospital of Zhengzhou University, Zhengzhou, 450052, P. R. China; ^4^ Division of Endocrinology and Metabolism, the Johns Hopkins University School of Medicine, Baltimore, MD 21287, USA

**Keywords:** thyroid cancer, CCDC67, tumor suppressor, prognostic marker, molecular pathogenesis

## Abstract

**Background:**

Some studies showed an association of coiled-coil domain-containing (*CCDC*) genes with cancers. Our previous limited data specifically suggested a possible pathogenic role of *CCDC67* in papillary thyroid cancer (PTC), but this has not been firmly established. The present study was to further investigate and establish this role of *CCDC67* in PTC.

**Results:**

The expression of *CCDC67*, both at mRNA and protein levels, was sharply down-regulated in PTC compared with normal thyroid tissues. Lower *CCDC67* expression was significantly associated with aggressive tumor behaviors, such as advanced tumor stages and lymph node metastasis, as well as *BRAF* mutation. Introduced expression of *CCDC67* in TPC-1 cells robustly inhibited cell proliferation, colony formation and migration, induced G1 phase cell cycle arrest, and increased cell apoptosis.

**Methods:**

Primary PTC tumors and matched normal thyroid tissues were obtained from 200 unselected patients at the initial surgery for detection of *CCDC67* mRNA and protein by RT-PCR and Western blotting analyses, respectively. Genomic DNA sequencing was performed to detect *BRAF* mutation in PTC tumors. Clinicopathological data were retrospectively reviewed for correlation analyses. PTC cell line TPC-1 with stable transfection of *CCDC67* was used to investigate the functions of CCDC67.

**Conclusions:**

This large study demonstrates down-regulation of *CCDC67* in PTC, an inverse relationship between *CCDC67* expression and PTC aggressiveness and *BRAF* mutation, and a robust inhibitory effect of *CCDC67* on PTC cellular activities. These results are consistent with *CCDC67* being a novel and impaired tumor suppressor gene in PTC, providing important prognostic and therapeutic implications for this cancer.

## INTRODUCTION

Thyroid carcinoma is a common endocrine malignancy that has seen a rapid increase in incidence globally in recent decades and is, in fact, currently the most rapidly increasing cancer among women and the second most among men [1, 2]. Papillary thyroid carcinoma (PTC) is the most common type of thyroid malignancy, accounting for over 90% of all thyroid malignancies [3]. Thyroid carcinoma is predominantly driven by genetic and epigenetic alterations, including activation of oncogenes and inactivation of tumor suppressor genes [4, 5]. Much still remains unknown about the molecular mechanisms of PTC even though many genetic and epigenetic alterations have been known in thyroid carcinoma, such as aberrant hypermethylation of CpG islands in promoter regions of tumor suppressor genes (TSGs) [6, 7].

The coiled-coil domain-containing (*CCDC*) proteins exhibit diverse functions related to their highly versatile folding motif [8]. The coiled-coil motif is found in many proteins, such as skeletal and motor proteins, and is involved in molecular recognition systems and protein refolding [9]. Previous studies have shown genetic or epigenetic alterations in several *CCDC* genes in human cancers, including *CCDC19* (NESG1) in nasopharyngeal carcinoma [10], *CCDC62* in prostate cancer [11], *CCDC116* in pancreatic cancer [12], *CCDC6* in lung cancer [13], *CCDC8* in renal carcinoma [14], *CCDC98* in breast cancer [15], and *CCDC134* in gastric cancer [16]. *CCDC67* is located in chromosome 11q21 and encodes a 604 amino acid protein containing a coiled-coil domain, including 3B79 (GenBank accession no.CG464599) clone sites with NotI/EcoRV segments covering the 5 'upstream area, which are associated with cell signal transduction in tumor formation [17, 18]. Park [18] found down-regulation and aberrant methylation of *CCDC67* in gastric cancer.

The role of *CCDC67* in thyroid carcinoma has not been well investigated except for a recent preliminary study on a limited cohort of patients in which we observed a down-regulation of *CCDC67* mRNA in PTC and an association of this down-regulation with some aggressive features of PTC [19]. Although this finding seems to suggest that *CCDC67* is a novel tumor suppressor gene in PTC, no firm conclusion could be established due to the limited scope of the study. Functional data is also lacking regarding a tumor suppressor role of this gene in PTC. In the present study on a large cohort of patients, we explored further to establish *CCDC67* as a novel tumor suppressor gene in PTC.

## RESULTS

### Down-regulation of *CCDC67* expression in PTC and tumor aggressiveness

**Figure 1:**
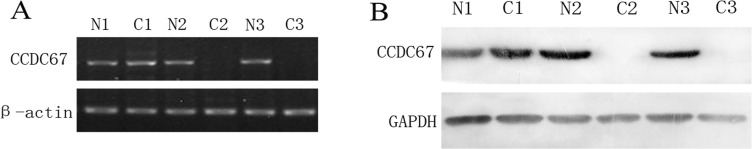
Expression status of *CCDC67* in PTC tumors (C) and matched adjacent normal thyroid tissues (N) (**A**) Shown are examples, representing 200 patients with papillary thyroid cancer, of RT-PCR analysis of *CCDC67* mRNA expression in PTC and adjacent tissues. (**B**) Western blot analysis of *CCDC67* protein in PTC and adjacent normal tissues. N, matched normal thyroid tissue; C, cancer tissue.

To firmly establish the clinicopathological role of *CCDC67* in PTC, here we dramatically expanded the cohort of patients to 200 cases from our previous small cohort [19] and additionally examined protein expression of *CCDC67* (previously only mRNA was examined). RT-PCR and Western blot were used to detect the expression of *CCDC67* in 200 PTC samples and their matched adjacent normal thyroid tissues. Representative results are shown in Figure 1. As shown in Figure 1A, some cases showed expression of *CCDC67* mRNA and some did not. All the 200 matched normal tissues showed robust expression of *CCDC67* mRNA while only 47.0% (94/200) cases of PTC tumors expressed *CCDC67* mRNA expression. Similar pattern was seen with the expression of *CCDC67* protein (Figure 1B). All the 200 samples of normal tissues expressed *CCDC67* protein while only 41.5% (83/200) cases of their matched PTC tumors expressed *CCDC67* protein. There was a strong correlation between the mRNA and protein expression of the *CCDC67* gene (*χ*^2^ = 33.038, *P* < 0.01) (Table 1). Interestingly, lack of *CCDC67* mRNA expression was associated with more aggressive tumor stages and lymph node metastasis (Table 2). Specifically, a dramatically higher number of cases of PTC without *CCDC67* mRNA expression displayed high TNM stages III/IV, pathological grade II, and lymph node metastasis, with *χ*^2^ = 8.236, 4.236, and 4.322, respectively, and *P* < 0.05 in all cases. A similar significant association was seen between the lack of *CCDC67* protein expression and high TNM stages III/IV, pathological grade II, and lymph node metastasis, with *χ*^2^ = 3.860, 5.548, and 7.275, respectively, and *P* < 0.05 in all cases (Table 2).

**Table 1:** Relationship between mRNA and protein expression of *CCDC6*7

*CCDC67* protein	*CCDC67* mRNA		Total
	+	−	
+	59	35	94
−	24	82	106
Total	83	117	200

χ^2^ = 33.038, *P* < 0.01.

**Table 2:** The relationship between *CCDC67* expression and clinicopathological features in PTC

Clinical date	Expression of mRNA		*P*	Expression of protein		*P*
	Positive	Negative		Positive	Negative	
Sex						
male	37	47	0.477	34	50	0.803
female	57	59		49	67	
Age						
< 45	40	56	0.147	36	60	0.270
≥ 45	54	50		47	57	
Tumor size						
< 2 cm	57	51	0.076	49	59	0.229
≥ 2 cm	37	55		34	58	
TNM stage						
I~II	59	45	0.004	50	54	0.049
III~IV	35	61		33	63	
Pathological grade						
I	58	50	0.040	53	55	0.019
II	36	56		30	62	
Lymph node metastasis						
NO	59	51	0.038	55	55	0.007
YES	35	55		28	62	
Total	94	106		83	117	

### Association between the down-regulation of *CCDC67* expression and the presence of BRAF T1799A mutation

**Figure 2:**
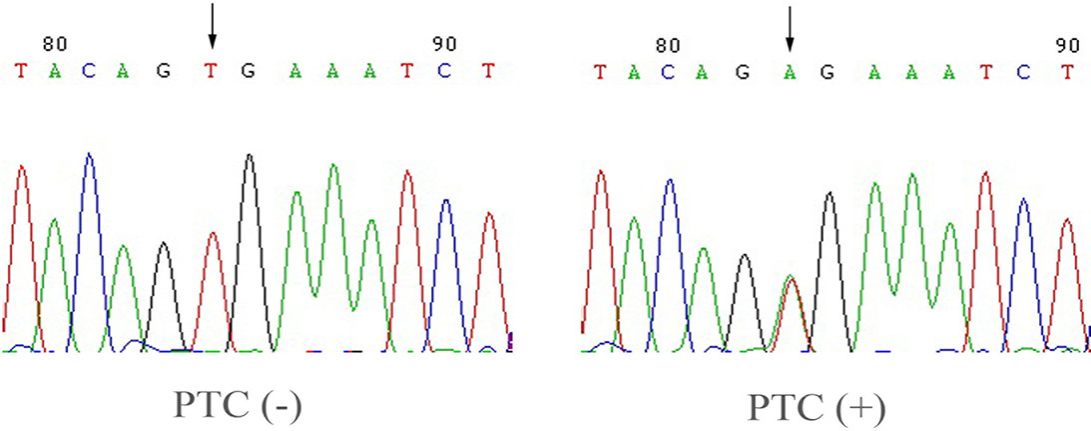
Detection of BRAF T1799A in PTC Shown is the representative electropherogram of the hot spot in exon 15 of the *BRAF* gene carrying the BRAF T1799A mutation. A case of PTC with the wild-type *BRAF* (PTC–) and a case with the BRAF T1799A mutation (PTC+) are shown. The arrow indicates the nucleotide where the mutation occurs.

One hundred sixty-eight of the 200 PTC tissues were available for genomic DNA isolation and analysis for *BRAF* T1799A mutation. The *BRAF* mutation was found only in tumor samples but not in their normal counterparts. Figure 2 shows a representative electropherogram of the *BRAF* 1799A mutation. We found that 42.3% (71/168) cases of PTC expressed *CCDC67* mRNA and 39.3% (66/168) cases of PTC harbored the *BRAF* mutation. There was a strong association between the down-regulation of *CCDC67* expression and the presence of the *BRAF* mutation (*χ*^2^ = 17.000, *P* < 0.01, Table 3).

**Table 3:** Relationship between *CCDC67* mRNA expression and *BRAF* mutation

*BRAF* mutation	*CCDC67* mRNA		Total
	+	−	
+	15	51	66
−	56	46	102
Total	71	97	168

χ^2^ = 17.000, *P* < 0.01.

### Overexpression of *CCDC67* suppressed TPC-1 cell proliferation and colony formation

**Figure 3:**
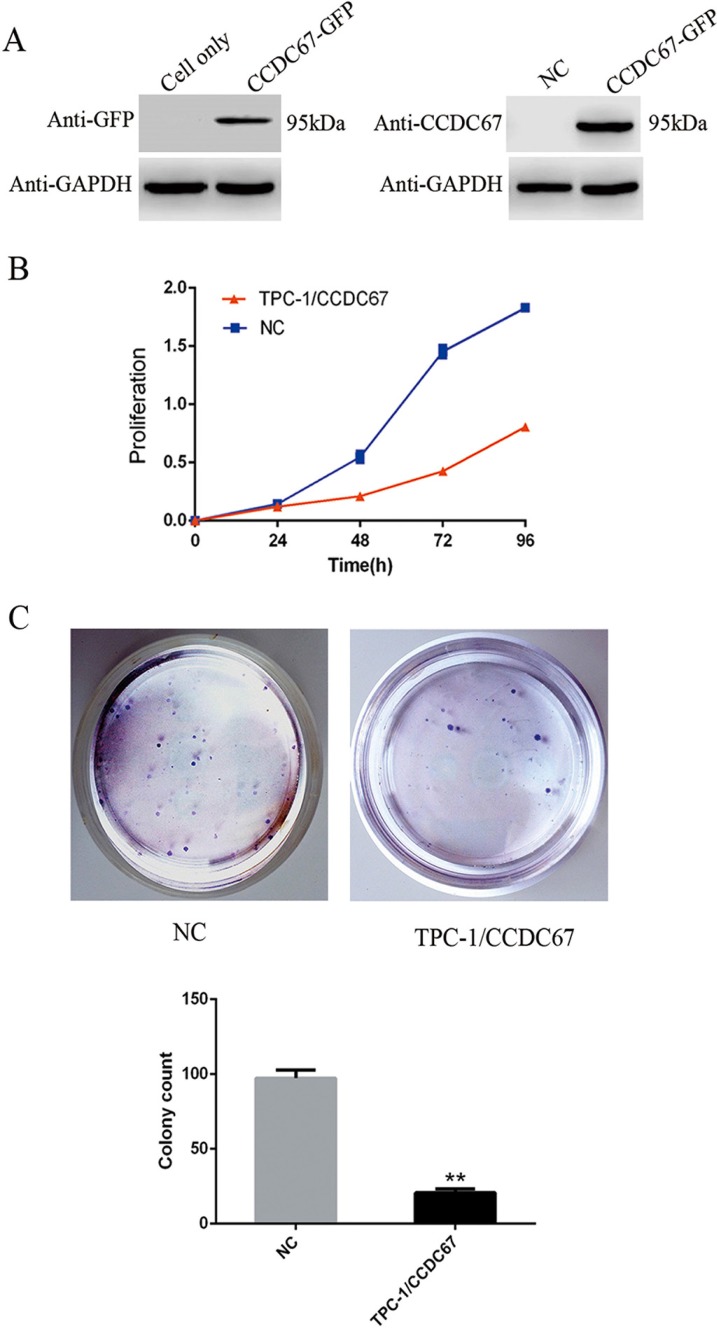
Introduced overexpression of *CCDC67* suppressed TPC-1 cell proliferation and colony formation (**A**) Introduced expression of *CCDC67* in transfected PTC-1 cells. TPC-1 cells, which naturally lacked *CCDC67* expression, were transfected with the *CCDC67*-GFP expressing vector or the empty vector as control (NC). Western blotting was performed using anti-GFP or anti-*CCDC67* antibodies. GAPDH protein was tested as a control. (**B**) Cell proliferation assay. TPC-1 cells expressing *CCDC67* or control cells (NC) were cultured in 96-well plates for the indicated time (1–4 days), and cell proliferation was measured using a CCK-8 kit. Proliferation was significantly decreased in TPC-1 cells expressing *CCDC67* compared with NC. (**C**) Adhesion-dependent colony-formation assay in monolayer culture. TPC-1 cells expressing *CCDC67* and NC cells were plated in six-well plates at 2000 cells/well. After 2 weeks of incubation, cells were stained with 0.1% crystal violet. Representative images are shown. In the bar graphs, the columns represent the mean colony number from three independent experiments and the little vertical bars on the top of the columns represent SD. **P < 0.01 in comparison with the control per Student's *t* test.

The above data strongly support *CCDC67* being a tumor suppressor gene. To further test this, we used TPC-1 cells, which naturally lack expression of *CCDC67* as we previously demonstrated [19] and as shown in Figure 3A, to examine the effect of introduced expression of *CCDC67* on cellular activities. Stably transfected *CCDC67*-expressing TPC-1 cells were established using a GFP-tagged *CCDC67* expression vector (Figure 3A). The effect of *CCDC67* on cell proliferation was examined using the CCK-8 assay and a significant inhibition of cell proliferation by induced *CCDC67* expression compared with the control (transfection with empty vector (NC) was observed (Figure 3B). Cell colony formation was also significantly inhibited by *CCDC67* overexpression (Figure 3C).

### *CCDC67* inhibited the migration and invasion of TPC-1 cells

**Figure 4:**
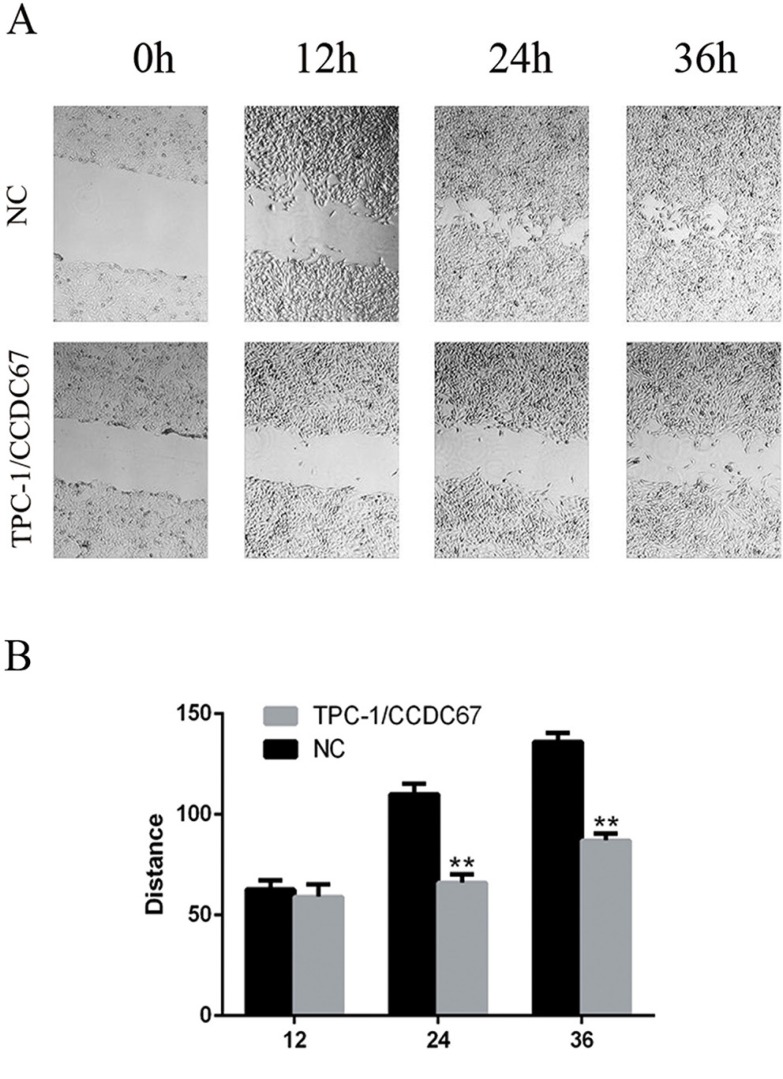
Wound healing assay of TPC-1 cell migration (**A**) Representative images of wound healing (cell migration) as described in the Methods in TPC-1 cells with induced overexpression of *CCDC67* (TPC-1/*CCDC67*) and control TPC-1 cells transfected with the empty vector (NC). Suppression of wound healing by *CCDC67* is clearly visible. (**B**) Bar graph presentation of quantitative measurements of cell migration distance in PTC-1 cells over-expressing *CCDC67* (TPC-1/*CCDC67*) and control cells (NC). Columns represent the mean of at least 3 independent experiments and the little vertical bars at the top of the columns represent SD. **P < 0.01 in comparison with the control per Student's *t* test.

**Figure 5:**
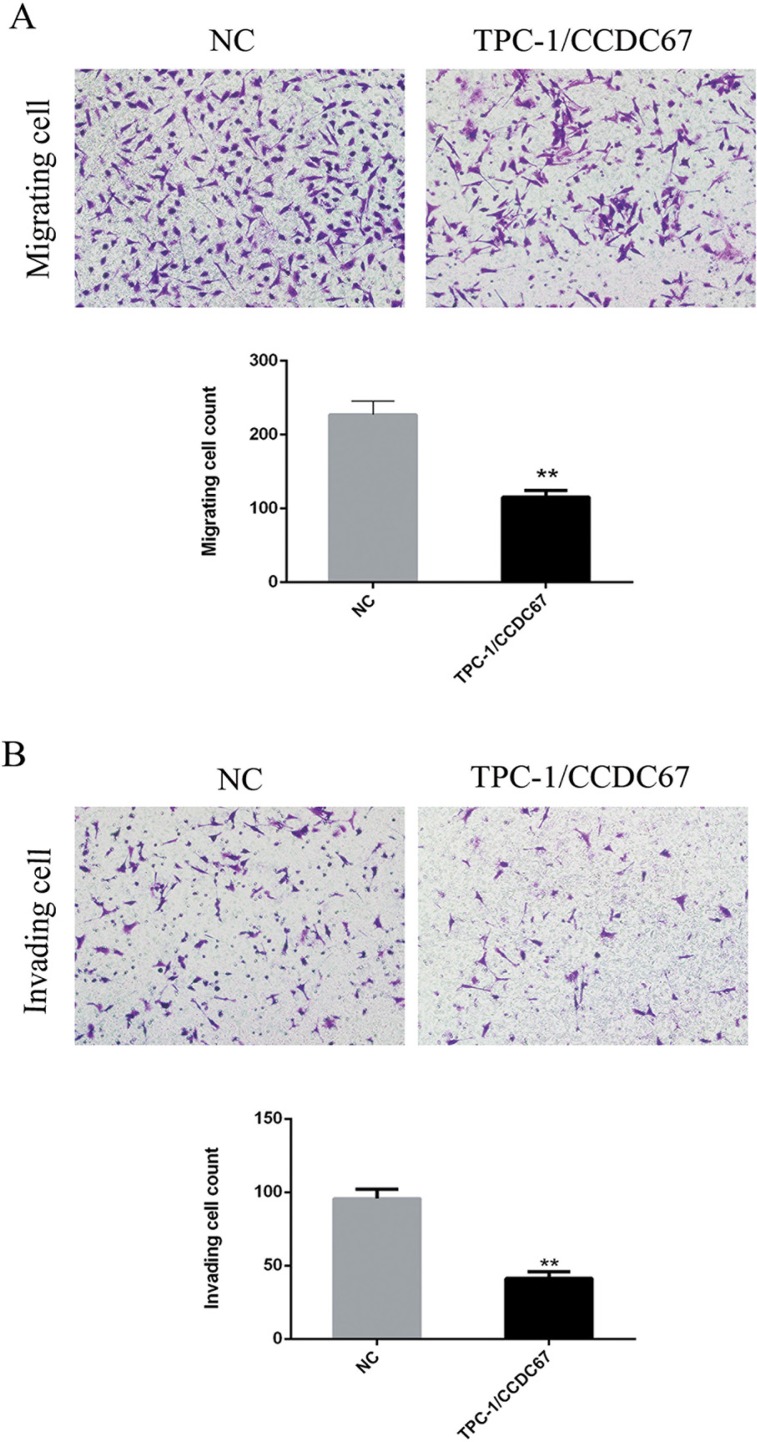
Migration and transwell invasion assays of TPC-1 cells (**A**) Migration assay. Shown in the top panel are representative microphotograph images of migrating TPC-1 cells transfected with the empty vector (NC) or cells with stably introduced expression of *CCDC67* (TPC-1/*CCDC67*). *CCDC67* (TPC-1/*CCDC67*) cells had a reduced ability to cross the transwell membrane compared to NC cells as demonstrated by the weaker staining of the transwells in which they were seeded. Shown in the lower panel is the bar graph presentation of quantitative measurements of cell migration in control TPC-1 cells (NC) and TPC-1 cells over-expressing *CCDC67* (TPC-1/*CCDC67*). Columns represent the mean of the counts of migrating cell numbers from at least 3 independent experiments and the little vertical bars at the top of the columns represent SD. (**B**) Invasion assay. Shown in the top panel are representative microphotograph images of invading TPC-1 cells transfected with the empty vector (NC) or cells with stably introduced expression of *CCDC67* (TPC-1/*CCDC67*). *CCDC67* (TPC-1/*CCDC67*) cells had a reduced ability to cross the transwell membrane with Matrigel matrix compared to NC cells as demonstrated by the weaker staining of the transwells in which they were seeded. Shown in the lower panel is the bar graph presentation of quantitative measurements of cell invasion in control TPC-1 cells (NC) and TPC-1 cells over-expressing *CCDC67* (TPC-1/*CCDC67*). Columns represent the mean of the counts of invading cell numbers from at least 3 independent experiments and the little vertical bars at the top of the columns represent SD. **P < 0.01 in comparison with the control per Student's *t* test in both panels A and B.

We next tested whether *CCDC67* could inhibit the migratory and invasive abilities of TPC-1 cells by performing the wound healing (cell migration) assay and the migration and transwell invasion assays. The wound healing ability (migratory ability) of cells was significantly decreased in TPC-1 cells with introduced overexpression of *CCDC67* compared with the control cells transfected only with the empty vector (Figure 4A and Figure 4B). The migration and transwell invasion assays showed that *CCDC67* overexpression also significantly suppressed the migration (Figure 5A) and invasion (Figure 5B) of TPC-1 cells as evidenced by a significant decrease in the number of migrating and invading cells, respectively (both *P* < 0.01).

### Overexpression of *CCDC67* induced G1 cell cycle arrest and promoted apoptosis of TPC-1 cells

**Figure 6:**
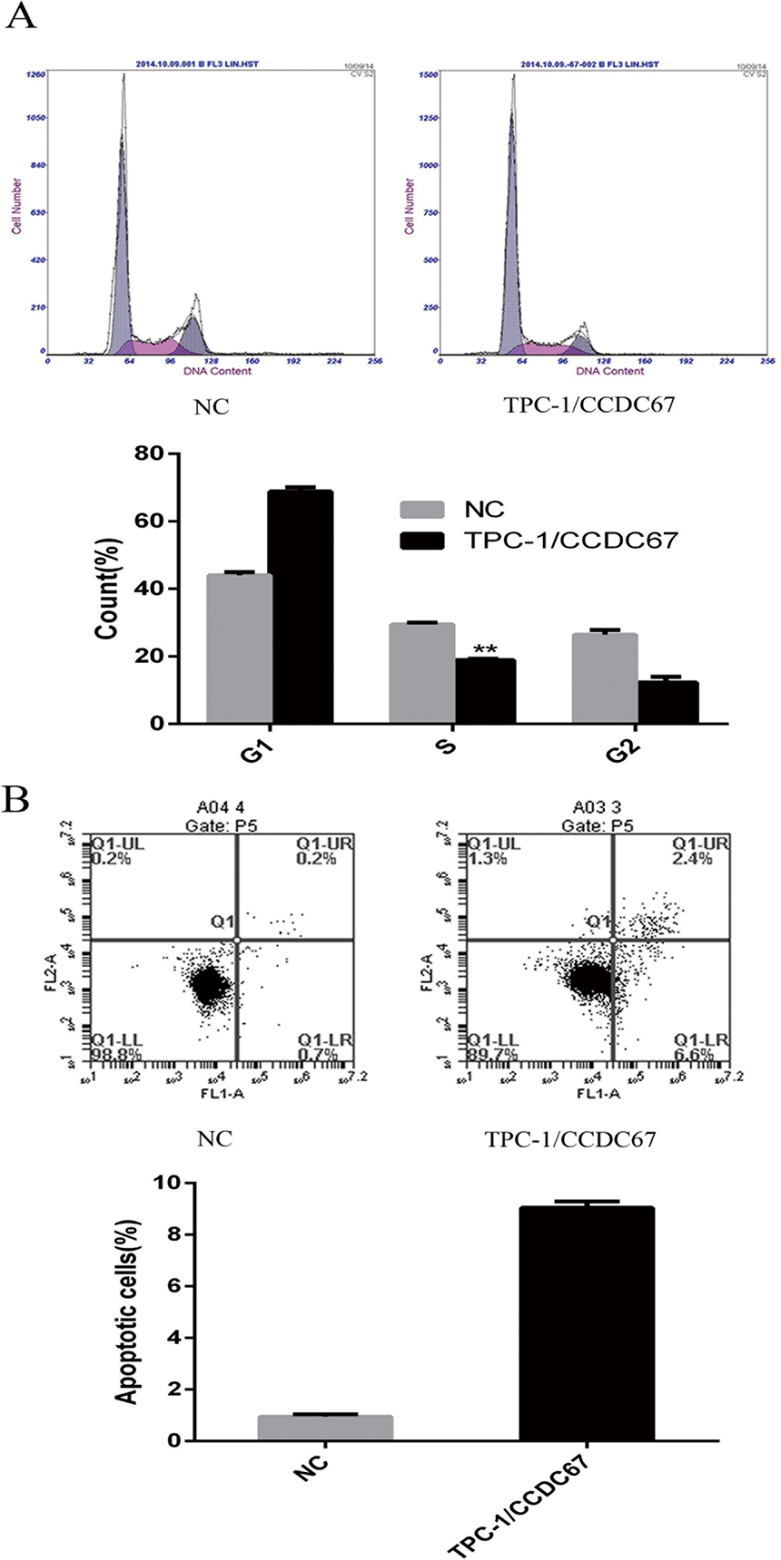
Effects of *CCDC67* on cell cycle and apoptosis of TPC-1 cells (**A**) Compared with the control cells (NC), TPC-1 cells with introduced expression of *CCDC67* (TPC-1/*CCDC67*) displayed a significant increase in the G1 phase and decrease in the S phase in the cell cycle. The upper panel shows the distribution of cell cycle phases and the lower panel shows the quantitative measurements of the cell cycle phases. (**B**) Introduced expression of *CCDC67* promoted cell apoptosis. The upper panel shows the extent of the apoptotic component of cells and the lower panels showed the quantitative measurements of the apoptotic cells. In the bar graphs, the columns represent mean of at least 3 independent experiments and the little vertical bars on the top of the columns represent SD. **P < 0.01 in comparison with controls per Student's *t*-test.

We also examined the effect of *CCDC67* on cell cycle distribution using flow cytometry. As shown in Figure 6A, compared with the control, introduced expression of *CCDC67* in TPC-1 cells promoted a significant shift of the cell cycle to the G0/G1 phase from the S phase. The apoptosis rate of cells was also significantly increased by introduced expression of *CCDC67* (Figure 6B).

## DISCUSSION

The coiled-coil motif of proteins is important for molecular recognition in the regulation of cellular functions and activities, through which these proteins may play an important role in human tumorigenesis [20–24]. One of such proteins, *CCDC67*, was previously indicated to play a possible suppressor role in tumor metastasis [25]. The promoter of the *CCDC67* gene was found to be hypermethylated in gastric cancer [18]. This is consistent with *CCDC67* being a tumor suppressor gene since, in addition to genetic impairment, such as genetic deletion and loss-of-function mutations, epigenetic alteration in the form of DNA methylation of the promoter regions is also an important mechanism in the inactivation of tumor suppressor genes [26–28]. The tumor suppressor role of the *CCDC67* gene, however, has not been firmly established. Its role in thyroid tumorigenesis is completely unknown. In the present study we investigated the potential tumor suppressor function of *CCDC67* specifically in thyroid cancer.

In the present analysis of a large cohort of 200 patients with PTC, we demonstrated a down-regulation of *CCDC67* expression in PTC tumors compared with normal thyroid tissues. We also demonstrated an association between tumor aggressiveness and decreased expression of *CCDC67*. These results were consistent with *CCDC6*7 being a tumor suppressor gene in PTC, which prompted us to take the next step to functionally study the *CCDC67* gene in PTC cells. Indeed, using a variety of cellular and molecular approaches, we demonstrated a remarkable suppressing effect of introduced expression of *CCDC67* on several cellular activities, such as cell proliferation, colony formation, migration, invasion, and tumor spheroid formation. These results are all well consistent with *CCDC67* being a tumor suppressor gene in thyroid cancer. Our finding of the strong pro-apoptotic function of *CCDC67* is in line with the recent report that CCDC proteins play a role in the cellular apoptotic process [29]. The role of *CCDC67* in thyroid cancer cellular activities observed in the present study is likely influenced by the microenvironments as studies in recent years have demonstrated that the latter play a critical role in thyroid cancer cell behavior [30–32].

The molecular pathogenesis of thyroid cancer involves genetic alterations in both oncogenes and tumor suppressor genes, as exemplified by *BRAF* and *RAS* for the former and *PTEN* [1] and *RASAL1* [33] for the latter. Our finding in the present study of the association between down-regulation of *CCDC67* and *BRAF* mutation in PTC suggests that the latter might play a role in the negative regulation of the former. Identification of *CCDC67* as a novel tumor suppressor gene in thyroid cancer expands the genetic repertoire for thyroid cancer. The specific mechanisms involved in the tumor suppressor role of *CCDC67* and whether there is genetic or epigenetic alteration in the *CCDC67* gene in thyroid cancer remains to be investigated. Nevertheless, the clinicopathological data and the cellular functional data in the present study are sufficient to establish that *CCDC67* is a novel tumor suppressor gene in thyroid cancer. Adding to our understanding of the molecular pathogenesis of thyroid cancer, these findings have important biological and clinical implications for this cancer.

## MATERIALS AND METHODS

### Patients and tissue samples

Two hundreds primary PTC samples and matched adjacent normal thyroid tissue samples were obtained at the time of initial surgery and snap-frozen immediately after tumor removal. The study was approved by the ethics committee of Zhengzhou University and human tissues were obtained with informed written consents from the patients. All tissue samples were reviewed by an endocrine pathologist to confirm the diagnosis. PTC samples estimated to contain more than 80% tumor cells were used.

### Cell lines and cell culture

The TPC-1 cell line was kindly provided by Dr Ye Lei of Shanghai Rui Jin Hospital, Shanghai, China. This cell was cultured in RPMI1640 supplemented with 10% fetal bovine serum and incubated at 37°C in a humidified atmosphere containing 5% CO_2_.

### RT-PCR analysis

Total RNA was extracted from PTC tissues and cell lines using the Trizol reagent (Invitrogen, USA). Total RNA (1 μg) was reverse-transcribed to cDNA using PrimeScript™ II kit (Takara, Japan) according to the manufacturer' instructions. One μl of the resulting cDNA was used as the template for PCR in a 25-μl reaction volume containing 12.5 μl Premix Taq (Takara, Japan), 1 μl of each primer, and 5 μl ddH_2_O. The β-actin mRNA level was used for normalization. PCR primer sequences are as follows—for β-actin: 5′-CTAAGTCATAGTCCGCCTAGAAGCA-3′(forward) and 5′-TGGCACCCAGCACAATGAA-3′(reverse); for *CCDC67*: 5′-GCAGCTCTGAAATTCCTCGT-3′ (forward) and 5′-TTGGTTGATCTTGCATCACTG-3′ (reverse). The PCR cycles were performed as follows: one cycle at 95°C for 3 min, 40 cycles at 95°C for 30s, 60°C for 30s and 72°C for 30s, and one final cycle at 72°C for 10 min, followed by cooling to 4°C. Amplification products were separated on 3% agarose gels and photographed with a UV gel imaging system (Kodak, USA). Imagemaster DVS system was used to determine the relative mean gray values (A) of the target product and β-actin. Expression index (I) of the target product was calculated using the formula of I = A product/A β-actin as previously described [34].

### Detection of *BRAF* mutation in PTC

The *BRAF* T1799A mutation was analyzed on genomic DNA isolated from 168 cases of primary PTC tissue samples that were available for DNA isolation. DNA was extracted from frozen tissue with the TaKaRa MiniBEST Universal Genomic DNA Extraction Kit (TaKaRa, Japan), according to the manufacturer's instructions.

*BRAF* gene mutation was detected by direct genomic DNA sequencing analysis. To this end, a 212-bp fragment from exon 15 containing the site where T1799A mutation occurs was amplified by PCR. The primers used were as described previously [35]: TCATAATGCTTGCTCTGATAGGA (forward) and GGCCAAAAATTTAATCAGTGGA (reverse). The amplification PCR was performed with deoxynucleotides using a step-down protocol: 98°C for 10s, 55°C for 15s, and 68°C for 1 min for 30 cycles. The reaction mixture contained about 80 ng genomic DNA, 10 μl 5 × PrimeSTAR GXL Buffer, 4 μl dNTP Mixture (2.5 nM), 31 μl ddH_2_O, 1 μl of each primer (forward and reverse), and 1 μl PrimeSTAR GXL DNA polymerase(TaKaRa, Japan) in a 50 μl final volume. After confirmation of the efficiency and quality of the amplification PCR by running the PCR products on a 2.0% agarose gel, the PCR products were were purified by DNA Gel Extraction Kit (TaKaRa, Japan) and subjected to direct sequencing PCR using the above-described forward primer. The purified PCR products were sequenced by Lifetech Company in Shanghai, China.

### Western blot analysis

For Western blotting, 30 mg of fresh frozen tumor or the adjacent normal tissue or treated cells were lysed in RIPA lysis buffer (Cwbiotech, China) containing 1% Protease Inhibitor Cocktail (Cwbiotech, China). An equal amount of protein of about 30 μg was separated by sodium dodecyl sulfate polyacrylamide gel electrophoresis (SDS-PAGE) and transferred onto the poly vinylidene fluoride (PVDF) membrane. After blocking with 5% skimmed milk, the PVDF membrane was incubated with anti-*CCDC67* antibody (Abcam, USA). After washing three times with Tris-Buffered Saline and Tween 20, the membrane was incubated with horseradish peroxidase-linked secondary anti-mouse IgG antibody (Abcam, USA) at room temperature for 1 h, followed by visualization using an ECL detection kit (Millipore, Billerica, MA). GAPDH protein, detected using an anti-GAPDH antibody purchased from Abcam, was used for loading control. Imagemaster DVS system was used to determine the relative mean gray values (A) of the target product and GAPDH. Expression index (I) of the target product was calculated using the formula of I = A product/A GAPDH [34].

### Construction of lentivirus expression vector of *CCDC67* and cell transfection

To construct the lentivirus vector expressing *CCDC67*, we amplified a cDNA fragment containing *CCDC67* precursor from TPC-1 cells (forward: 5′-GAGGATCCCCGGGTACCGGTCGCCACCATGGAGA ACCAAGCCCATAATAC-3′, reverse: 5′-TCACCATGGTG GCGACCGGTATGTGTCTATTTTGTTTTAGC-3′). The amplified fragment was cloned into a modified pGV208-GFP vector (Genechem, China). A day before lentivirus infection, TPC-1 cells in normal culture condition were seeded into six-well culture plates and grown overnight. Cells were then transfected with the pGV208-GFP-*CCDC67* vector (defined as TPC-1/*CCDC67*) according to the multiplicity of infection (MOI = 1). The empty vector pGV208-GFP was used for negative control (defined as NC). After an incubation for 96 h, detection of the expression was performed under fluorescence.

### Cell proliferation assay and colony forming assay

For the proliferation assay, TPC-1/*CCDC67* and NC cells were seeded at 5 × 10^3^ cells/well in 96-well plates and incubated at 37°C for 4 days. An aliquot of 10 μl Cell Counting Kit-8 reagent (Dojindo, Japan) was added to the cells and, following a 3-h incubation, absorbance was measured at 450 nm using a spectrophotometer (Bio-Rad, USA).

For the colony forming assay, the two transfectant cells were plated at 100 cells/well in six-well plates, incubated for 2 weeks in RPMI-1640 containing puromycin (1 μg/ml), and stained with crystal violet. Colonies were photographed and counted after 3 weeks of incubation at 37°C. All assays were performed in triplicate.

### Migration and invasion assay

For migration assay, 200 μl of the TPC-1/*CCDC67* and NC cells in serum-free RPIM-1640 were seeded on upper migration chambers (24-well plates, 8–μm pore size. Corning Incorporated Costar, USA) at a density of 2 × 10^5^ cells/well, and incubated at 37°C. A volume of 600 μl of RPIM-1640 containing 10% fetal bovine serum was added in the lower chamber. Wound healing assay was also carried out to detect the migration of cells and the two transfectant cells were cultured in 6-well plates until confluent. The cell layer was wounded using a sterile tip. After incubation for 24 to 36 hours, cells were photographed under a phase-contrast microscope. Experiments were carried out in triplicate. The distance between the two edges of the scratch (wound width) was measured at 10 sites in each image. The cell migration distance was determined by measuring the wound width at each time point from the wound width at the 0 h time point and then dividing by two [36].

For invasion assay, Matrigel matrix (BD, USA) was dissolved at 4°C overnight and then diluted with RPIM-1640. The upper chambers were coated with the diluted solution (100 μl/well) and then placed at 37°C for 4 h to solidify the Matrigel matrix. The rest procedures were same with migration assay.

The migrating or invading cells in upper migration chambers were fixed with methanol for 15 minutes, and then stained with 0.1% crystal violet for 30 minutes. The unpenetrated cells on upper chamber were gently scraped from the surface with cotton swabs. Five visual fields (200×magnification) were selected for each chamber by Image J software (Nikon, Japan) and the number of penetrated cells in these five fields (200×magnification) was counted under an inverted microscope. Mean values of groups were recorded.

### Cell cycle analysis

TPC-1 cells transfected with *CCDC67* and control cells were harvested 72 h after culture, washed with cold phosphate buffered saline (PBS), and fixed in 1 ml of 70% ethanol. After overnight incubation at 4°C in ethanol, cells were washed in PBS and suspended in 500 ml propidine iodide (PI) 30 min before flow cytometry. Populations in G1, S, and G2 phases were measured by flow cytometry (Beckman, CA) and the data were analyzed using the Multicycle-DNA Cell Cycle Analyzed Software. The measurement was performed in triplicate.

### Detection of cell apoptosis

TPC-1 cells transfected with *CCDC67* and control cells were collected and fixed with 70% ethanol for the detection of apoptosis. The dyeing of cells was performed according to the instructions of Annexin V-FITC/PI cell apoptosis detection kit (SouthernBiotech, USA). Flow cytometry (BD Biosciences, USA) was used to detect the percentage of early apoptosis. The measurement was performed in triplicate.

### Statistical analysis

The results are expressed as mean ± the standard deviation (SD) of the mean of at least three separate experiments. Statistical significance was assessed using a two-tailed unpaired Student's *t*-test. The correlation between gene expression and potential causative variables were evaluated with the Chi-square test. *P* < 0.05 was considered to indicate a statistically significant result. The statistical analyses were performed using the SPSS 17.0 software for Windows. GraphPad Prism 5 (GraphPad Software, USA) was used for graphs.
